# Hydrothermal Carbonization of Heavy Metal-Contaminated Biomass: Migration, Transformation, and Ecological Stability Changes of Metals

**DOI:** 10.3390/ijms26062551

**Published:** 2025-03-12

**Authors:** Jieni Wang, Shuqin Zhang, Chenlin Wei, Haodong Hou, Guozhen Song, Leichang Cao, Jinglai Zhang

**Affiliations:** 1 Henan Key Laboratory of Protection and Safety Energy Storage for Light Metal Materials, College of Chemistry and Molecular Sciences, Henan University, Kaifeng 475004, China; jieniwang@126.com (J.W.); zhangshuqin@henu.edu.cn (S.Z.); wcl2000@henu.edu.cn (C.W.); houhd@henu.edu.cn (H.H.); songgz@henu.edu.cn (G.S.); 2 Miami College, Henan University, Kaifeng 475004, China

**Keywords:** hydrothermal carbonization (HTC), heavy metals, migration and transformation, ecological stability, biochar

## Abstract

Developing effective treatment technologies for heavy metal-contaminated biomass is of great environmental significance. This study explores the hydrothermal carbonization (HTC) of biomass contaminated with heavy metals (Cu, Zn, Cd, and Pb), focusing on the migration, transformation, and ecological stability of these metals during the process. Biomass samples were treated under subcritical conditions at varying temperatures (170–260 °C) and reaction times (1–4 h). Results showed that heavy metals were mainly enriched in biochar (>98%), and Cu predominantly transformed into metallic copper (Cu^0^), Zn tended to form stable organometallic complexes or remain in non-volatile forms, Pb coexisted in both metallic and carbonate species, and Cd converted into metallic and oxidized states. The transformation of these metals was influenced by reaction parameters, such as temperature and time, which affected both their immobilization and the structural properties of the prepared hydrochar. The Tessier extraction experiments showed that the unstable state (F1, F2) of heavy metals in hydrochar was obviously reduced from 17.9% to 6.8%, and the heavy metals were significantly stabilized compared with the original biomass. This research highlights the potential of HTC as a dual-purpose technology for biomass conversion and heavy metal remediation, offering insights for stabilizing contaminants and producing environmentally stable biochar products.

## 1. Introduction

Due to industrial activities, agricultural runoff, and improper waste disposal, the accumulation of heavy metals such as copper (Cu), zinc (Zn), cadmium (Cd), and lead (Pb) in the environment is becoming more and more serious [1], causing severe ecological and human health risks [2]. Simultaneously, with the increasing application of biomass materials for heavy metal remediation (adsorption, stabilization, biotransformation, and phytoremediation) [3,4], heavy metal-contaminated biomass is growing as an environmental concern, making its direct utilization for bioenergy or other purposes challenging. Traditional biomass treatment methods primarily include landfill, incineration, and composting [5]. However, these methods often face challenges such as high energy cost, low efficiency, inefficient heavy metal separation, and a high risk of secondary pollution. The emerging advanced biomass treatment technologies include pyrolysis gasification, anaerobic fermentation, and hydrothermal conversion [6]. Amongst these, hydrothermal carbonization (HTC) has garnered significant attention due to its unique advantages. Unlike other methods, hydrothermal technology can directly process wet biomass without requiring additional drying pretreatment. It not only reduces the generation of harmful substances but also stabilizes pollutants and significantly enhances the energy content of biomass [7,8]. HTC is a thermochemical conversion technology that transforms biomass into carbon-rich solids, known as hydrochar, under moderate temperatures (180–260 °C) and pressures (2–10 MPa). Hydrothermal technology has emerged as a highly promising solution in the fields of biomass resource utilization and heavy metal pollution remediation [9].

As an environmentally sustainable process, HTC offers an opportunity to treat metal-contaminated biomass, but the behavior of heavy metals during HTC is still not fully understood. Specifically, the migration, transformation, and ecological stability of the metals during hydrothermal carbonization requires further exploration to optimize HTC for both pollutant removal and biomass valorization. In the process of optimizing hydrothermal carbonization to achieve efficient pollutant removal and biomass stabilization, the following key measures can be implemented to enhance treatment efficiency, environmental safety, and promote the efficient utilization of biomass resources: Optimization of reaction conditions: by systematically regulating key parameters such as reaction temperature, pressure, and reaction time, the efficient fixation of heavy metals and the maximization of biomass carbonization can be achieved. Introduction of chemical additives: the addition of chemical reagents such as catalysts, adsorbents, and chelating agents can adjust the chemical environment of the reaction system, promoting the precipitation, adsorption, or complexation of heavy metals, thereby reducing their mobility and bioavailability. Enhancement of the product post-treatment: physical or chemical modification of hydrochar can be performed to improve its adsorption performance, while deep treatment of liquid-phase products can be conducted to further recover or immobilize residual heavy metals. Despite the promising potential of HTC, several challenges remain in the treatment of heavy metal-contaminated biomass. First, while the process is known to reduce the bioavailability of heavy metals by transforming them into more stable forms [10], the specific mechanisms of metal migration and transformation under various HTC conditions (e.g., temperature, pressure, and reaction time) have not been fully elucidated. Heavy metals in biomass undergo various chemical changes, such as oxidation [11], reduction [12], and complexation [13], but how these processes are influenced by different operational parameters remains poorly understood. Furthermore, the stability of heavy metals in the prepared hydrochar, particularly in terms of leaching and long-term environmental impact, needs more in-depth investigation.

Additionally, existing research mostly focuses on either the carbonization efficiency or the metal stabilization capacity of HTC (Table S1, Supplementary Materials). Yadav et al. investigated the hydrothermal liquefaction kinetics and product characteristics of copper-impregnated water hyacinth, with a particular focus on the effects of reaction time on the yields of bio-oil, biochar, aqueous-phase products, and gas [14]. However, the study did not explore the feasibility and economic viability of these products in practical applications, which limits its practical relevance. Stobernack et al. examined the effects of a multi-stage process combining hydrothermal carbonization and thermochemical treatment on the migration of phosphorus and heavy metals in sludge [15]. While the study provided valuable insights into the migration behavior of these elements during sludge treatment, it was constrained by the use of single experimental conditions and the absence of an economic analysis, which could have enhanced its applicability. Ro et al. conducted experimental research on the effects of hydrothermal carbonization at different temperatures on chicken manure and pig manure [16]. Lu et al. conducted a comprehensive investigation into the effects of hydrothermal carbonization conditions on heavy metal stabilization and fuel characteristics during the co-carbonization process of sludge and wood chips [17]. However, these studies often focus on specific aspects without considering the comprehensive influence of multiple operational parameters (e.g., temperature, reaction time, and metal concentration) on both biomass conversion and heavy metal behavior. In summary, most studies have failed to simultaneously consider the comprehensive effects of key reaction parameters such as temperature, reaction time, and metal concentration on biomass conversion and metal behavior. The study primarily focused on analyzing the physical and chemical properties, stability, and migration and transformation mechanisms of inorganic components in biochar. There is a gap in understanding how these variables interact and influence the final product’s quality, particularly in terms of biochar’s ecological stability and its potential use in environmental remediation. Addressing these gaps is critical for optimizing HTC and ensuring that it can effectively treat metal-contaminated biomass while enhancing biochar’s applications.

This study aims to fill these gaps by investigating the migration, transformation, and ecological stability of heavy metals (Cu, Zn, Cd, and Pb) during the hydrothermal carbonization of metal-contaminated biomass. Our research focuses on understanding how reaction parameters, such as temperature, reaction time, and initial metal concentration, influence the distribution and chemical forms of heavy metals in the hydrochar, and how these factors affect the stability of metals in the biochar matrix. The aims of this work lie in the following: (1) addressing the understanding of heavy metal behavior during HTC, particularly focusing on their migration and transformation, and (2) evaluating the ecological stability of the prepared hydrochar through Tessier extraction experiments under diverse environmental conditions. The study will provide insights into how temperature and time influence the formation of metal–carbon and metal–oxide complexes, which are critical for assessing the potential use of biochar for environmental applications, such as heavy metal immobilization or soil amendment. Furthermore, by evaluating the effects of metal concentration, this research will help optimize HTC conditions for both biomass conversion and metal stabilization. It also extends the current understanding of heavy metal immobilization by considering the catalytic role of metals in the HTC process.

## 2. Results and Discussion

### 2.1. Changes in the Yield and Elemental Composition of Hydrochar

Table 1 shows the yield and elemental composition of hydrochar under different conditions. With temperature increasing from 170 °C to 260 °C, the yield of hydrochar first decreased from 47.54% to 26.30%, then increased to 42.53%. This is because under low-temperature conditions (below 200 °C), the moisture and organic components (such as cellulose and lignin) in biomass are not completely decomposed, and the resulting hydrochar is a partially carbonized product. When the temperature rises (above 200 °C), most of the organic matter is converted into a stable carbon structure. With the release of volatile components, decomposition of organic constituents, and formation of carbon structures, the pore structure of hydrochar was significantly optimized, leading to a substantial enhancement in adsorption performance and a further increase in yield [18]. As the copper content increases (0–50 mg/g), the yield of hydrochar significantly increases (from 32.29% to 43.35%). This is due to the catalytic role of copper ions in the degradation of organic matter during the carbonization process, stabilizing the carbonization products. As the concentration of heavy metals increases, copper ions catalyzed the decomposition of cellulose, hemicellulose, lignin, small-molecule organic compounds, and tar-like substances, reducing the generation of low-molecular compounds and promoting the stabilization of the carbon skeleton, thereby significantly increasing the yield of hydrochar.

**Figure 1:**
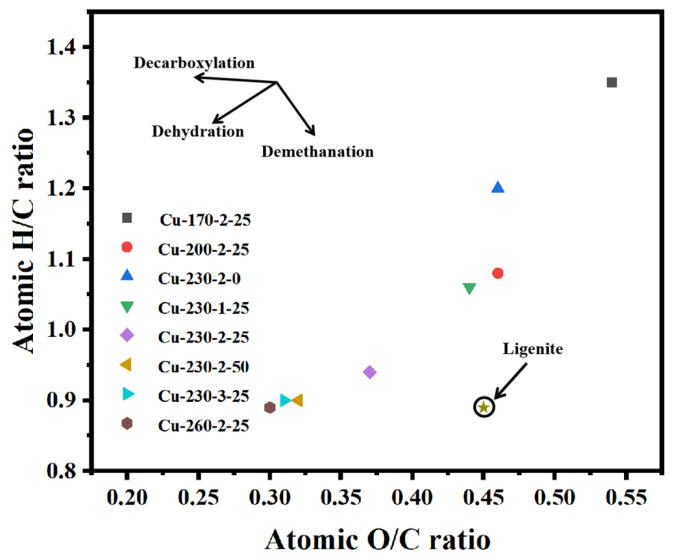
Van Krevelen diagram of hydrochar.

Figure 1 illustrates the van Krevelen diagram of hydrochar under different conditions. As the carbonization temperature increases, the O/C ratio of hydrochar decreases from 0.54 to 0.3, and the H/C atomic ratio decreases from 1.35 to 0.89. This indicates that during the HTC process, the aliphatic and carbohydrate structures in the biomass were gradually destroyed, forming a more stable aromatic carbon framework [19]. The aromatic carbon framework not only endowed hydrochar with higher stability, but also significantly improved its adsorption performance, conductivity, and pore structure. As shown in Figure 1, with the increase in copper content (0–50 mg/g), the O/C and H/C atomic ratios decrease from 0.46 and 1.2 to 0.32 and 0.9, respectively. This indicates that copper ions could promote the decomposition of oxygen-containing functional groups and facilitate dehydration and decarboxylation reactions [20].

### 2.2. Migration of Cu

#### 2.2.1. Effect of Temperature on Copper Migration

**Figure 2:**
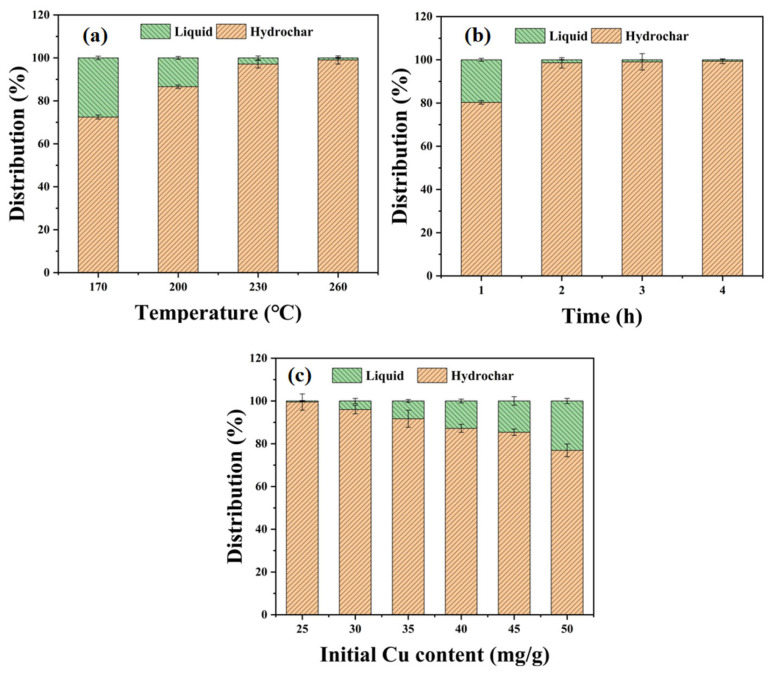
(**a**) The effect of temperature variation on Cu distribution under conditions of 3 h reaction time and an initial heavy metal concentration of 25 mg/g. (**b**) The effect of the reaction time on Cu distribution at a temperature maintained at 260 °C and with an initial heavy metal concentration of 25 mg/g. (**c**) The effect of an initial heavy metal concentration on Cu distribution under hydrothermal conditions of 230 °C and 2 h reaction time.

Figure 2a shows the effect of temperature on the distribution of copper in hydrochar (solid phase), with a fixed reaction time of 3 h and an initial Cu content of 25 mg/g. As the hydrothermal temperature increases from 170 °C to 260 °C, the copper distribution in hydrochar gradually increases from 72.7% to 99.2%, with the highest distribution being observed at 260 °C. This can be attributed to changes in both the aqueous phase and the hydrochar characteristics at higher temperatures, which facilitate metal migration and adsorption. The elevated carbonization temperature enhances the dissolution and migration of metal ions through modifications in the acidity, ligand composition, and dielectric properties of the aqueous phase. Simultaneously, the development of an aromatic carbon skeleton, evolution of pore architecture, and transformation of surface functionalities synergistically improve the metal adsorption capacity of hydrochar. This dual mechanism collectively accounts for the enhanced metal enrichment efficiency of hydrochar under high-temperature conditions. Higher temperatures can induce the cleavage and recombination of chemical bonds within hydrochar, resulting in a well-developed pore structure. Originally small pores may expand, and concurrently, some new pores will be generated, leading to an increase in the number of pores and an enlargement of the specific surface area. On the other hand, the pore size distribution will undergo changes, and mesopores or micropores conducive to the adsorption of metal ions may emerge. At high temperatures, the original oxygen-containing functional groups on the surface of hydrochar, such as hydroxyl groups (-OH) and carboxyl groups (-COOH), may decompose or transform, and some new oxygen-containing functional groups, such as carbonyl groups (C=O), are formed. These changes will alter the charge properties and chemical activity of the hydrochar surface. The increase in functional groups such as carbonyl groups may enhance the complexation ability of the hydrochar surface with some metal ions, thereby improving the adsorption performance. First, the specific surface area and pore structure of hydrochar are likely to change under high temperatures, enhancing its ability to adsorb metal ions. Additionally, the quantity and type of surface functional groups of hydrochar may change at higher temperatures, which alters its hydrophilicity or lipophilicity, further promoting heavy metal adsorption [21]. Furthermore, under high-temperature conditions, the rate of coordination and chemical reactions between heavy metals and the surface functional groups of hydrochar accelerates [22], enabling metal ions to migrate more efficiently from the aqueous phase to the hydrochar. As the carbonization temperature increases, the solubility of inorganic substances (e.g., carbonates and phosphates) decreases, resulting in an enhanced ability of these substances to bind with heavy metal ions and form precipitates [23], further promoting the immobilization of heavy metals. Elevated temperatures facilitate the formation of oxygen-containing functional groups (e.g., carboxylic, hydroxyl, phenolic hydroxyl, and ether groups) on hydrochar surfaces [23]. These functional groups significantly enhance heavy metal adsorption capacity through two primary mechanisms: chelate complexation with metal ions and electrostatic interaction-mediated binding.

#### 2.2.2. Effect of Reaction Time on Copper Migration

Figure 2b shows the effect of the reaction time on the distribution of copper in hydrochar under a fixed temperature of 260 °C and initial Cu content of 25 mg/g. It can be observed that as the reaction time increases from 1 h to 4 h, the copper distribution in hydrochar gradually increases from 80.1% to 99.8%, with the highest distribution observed at 4 h. This indicates that reaction time is an important factor controlling metal migration and adsorption during hydrothermal carbonization. As the reaction time is extended, the migration and immobilization of metals change. Firstly, as the reaction time increases, the adsorption sites on the hydrochar gradually become occupied by metal ions, resulting in a decrease in the metal ion concentration in the aqueous phase. Longer reaction times typically promote metal ion adsorption, allowing the metals to become more effectively fixed onto the hydrochar. Secondly, extended reaction times may enable more in-depth interactions between heavy metals in the aqueous phase and hydrochar, such as complexation, precipitation, and co-precipitation [3]. As the organic matter in hydrochar gradually converts into carbon, the adsorption capacity of the carbon-based material increases, thereby facilitating the fixation of more heavy metals [24]. In conclusion, appropriately increasing the reaction time promotes the migration and immobilization of heavy metals. However, an excessive reaction time may result in the saturation of adsorption sites and a reduction in the migration rate.

#### 2.2.3. Effect of Initial Cu Content on Copper Migration

Figure 2c shows the effect of initial Cu content on the distribution of Cu in hydrochar under a fixed temperature of 230 °C and a reaction time of 2 h. As the initial Cu content increases from 25 mg/g to 50 mg/g, the distribution of Cu in hydrochar gradually decreases, with the highest distribution being observed at 25 mg/g. This suggests that the initial concentration has a significant impact on metal migration during hydrothermal carbonization. At lower initial concentrations, the hydrochar can more easily fix heavy metals via adsorption. However, at higher initial concentrations, the adsorption sites on the hydrochar may be occupied by multiple metal ions [25], resulting in some ions being unable to be effectively immobilized, thereby influencing the migration of heavy metal ions to the solid phase. At this stage, the migration of heavy metals depends not only on temperature and pH, but also on the affinity between different metal ions and the availability of adsorption sites [26]. As the initial concentration further increases, the adsorption capacity of the hydrochar may reach saturation, leading to more metal ions being poorly immobilized or even desorbed back into the aqueous phase. In conclusion, high initial concentrations can lead to surface saturation of the hydrochar, influencing the migration and adsorption efficiency of metals. In some cases, higher initial concentrations may cause metal ions to precipitate or be adsorbed by hydrochar, affecting the migration process through competition with other ions.

In summary, high temperatures facilitate the dissolution and migration of metal ions, while also enhancing the adsorption capacity and reaction rate of hydrochar, thereby contributing to the immobilization of heavy metals. Longer reaction times promote more complete adsorption and immobilization of metal ions. However, excessive time may lead to the saturation of adsorption sites and slower migration. Higher initial concentrations may result in saturation and precipitation, influencing the hydrochar’s adsorption capacity and giving rise to competition between different metal ions. These factors collectively influence the migration and distribution of heavy metals during hydrothermal carbonization.

### 2.3. Transformation of Cu

#### 2.3.1. Chemical Speciation of Cu

**Figure 3:**
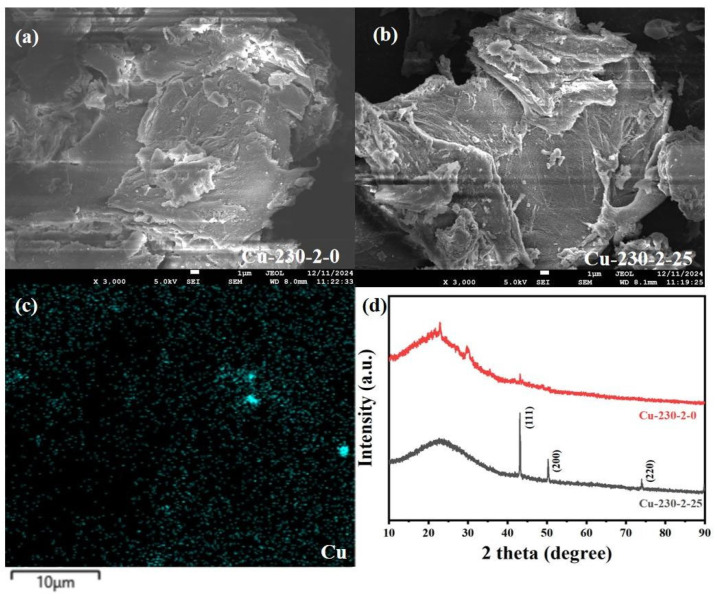
SEM analysis of hydrochar. (**a**) Hydrochar without added heavy metals, (**b**,**c**) hydrochar with added Cu, and (**d**) XRD analysis of hydrochar.

Figure 3a,b compare the microstructures of hydrochar derived from untreated biomass and biomass treated with Cu. Figure 3c shows the Cu-mapping image for Cu-containing hydrochar. As can be clearly observed from Figure 2a–c, the addition of copper results in the formation of numerous small spherical carbon particles on the surface of the hydrochar, with the overall surface structure becoming more developed, and Cu is uniformly distributed throughout the hydrochar. The presence of spherical carbon particles enhances the specific surface area of hydrochar. Moreover, the accumulation of these spherical carbon particles may also generate new pores between particles, which is conducive to the adsorption of heavy metals by hydrochar. Figure 3d presents the XRD analysis of the hydrochar without Cu and the Cu-loaded hydrochar (Cu-230-2-25). The positions, intensities, and shapes of the diffraction peaks verify the presence of copper and the basic carbon crystalline structure of the hydrochar. The XRD spectrum of Cu-loaded hydrochar discloses several key diffraction peaks, which confirm that copper exists in its elemental form. Based on the standard card [27], the diffraction peaks at 2θ values of 43.3°, 50.5°, and 74.1° correspond to the (111), (200), and (220) crystal planes of metallic copper [28]. These diffraction peaks indicate that elemental copper has been loaded onto the hydrochar in a crystalline state. Furthermore, the XRD pattern also reveals the typical peaks for the basic carbon structure of the hydrochar, with broad diffraction peaks around 2θ = 22° and 44°, corresponding to the (002) and (100) planes of carbon [29]. The peak at 2θ = 22° represents the amorphous carbon in the hydrochar, indicating its disordered structure [29]. Another relatively weak peak at 2θ = 44° corresponds to the (100) plane of carbon, suggesting local ordering within the carbon crystal. These diffraction peaks confirm that elemental copper is loaded onto the hydrochar, coexisting with the amorphous and microcrystalline carbon structures after the hydrothermal process.

#### 2.3.2. Ecotoxicity Changes of Cu

**Figure 4:**
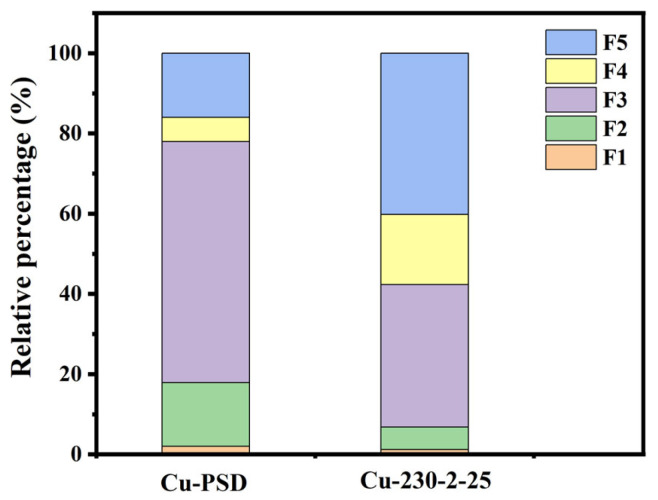
Ecological toxicity analysis of pine sawdust with Cu addition, and hydrochar derived from pine sawdust with Cu addition.

As shown in Figure 4, compared with the raw material, the ecological toxicity of Cu within the hydrochar product is significantly changed. The toxicity of the metal is reduced, primarily attributed to the transformation from unstable states (F1, F2) to more stable states (F4, F5). It may be attributed to the fact that the dissolved Cu^2+^ undergoes a chemical reaction with inorganic substances (such as FeO_x_, AlO_x_, and MnO_x_) present in the hydrochar, forming insoluble compounds [30]. The formation of these insoluble compounds reduces the bioavailability of heavy metals, thus decreasing their toxicity to ecosystems. For instance, Cu^2+^ is capable of forming insoluble copper oxides (CuO) under alkaline conditions, which reduces its absorption and accumulation in aquatic organisms. Copper may also form complexes with surface functional groups on the hydrochar [31], thereby lowering the concentration of free metal ions. Additionally, the porous structure of hydrochar and its surface functional groups (such as carboxyl and amino groups) provide strong adsorption sites for Cu ions. These ions are adsorbed onto the surface of the hydrochar, forming stable adsorbed states. This reduces their contact with biological systems, thereby decreasing ecotoxicity. This process generally leads to a significant reduction in the bioavailability of heavy metals.

### 2.4. Other Heavy Metals

**Figure 5:**
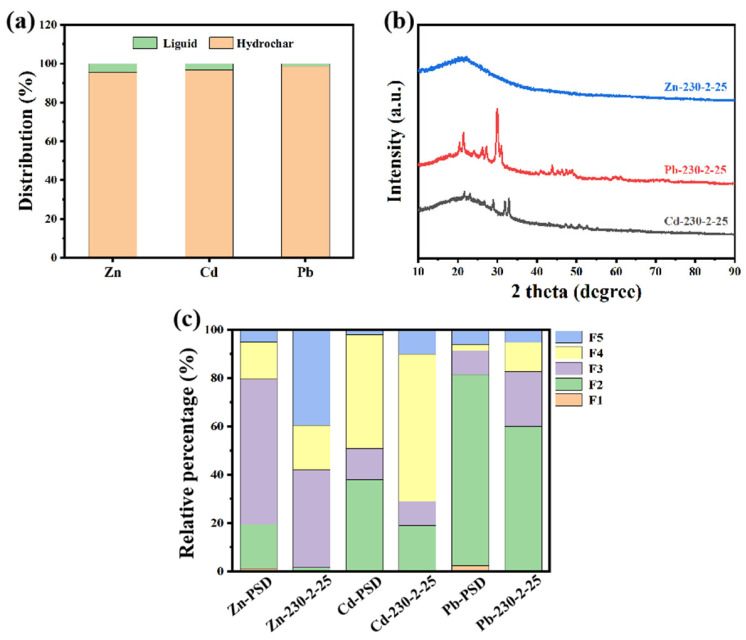
Migration and transformation analysis of different metals during hydrothermal carbonization process. (**a**) Distribution of different heavy metals in hydrothermal products, (**b**) XRD patterns of different heavy metals, and (**c**) ecotoxicity analysis.

**Figure 6:**
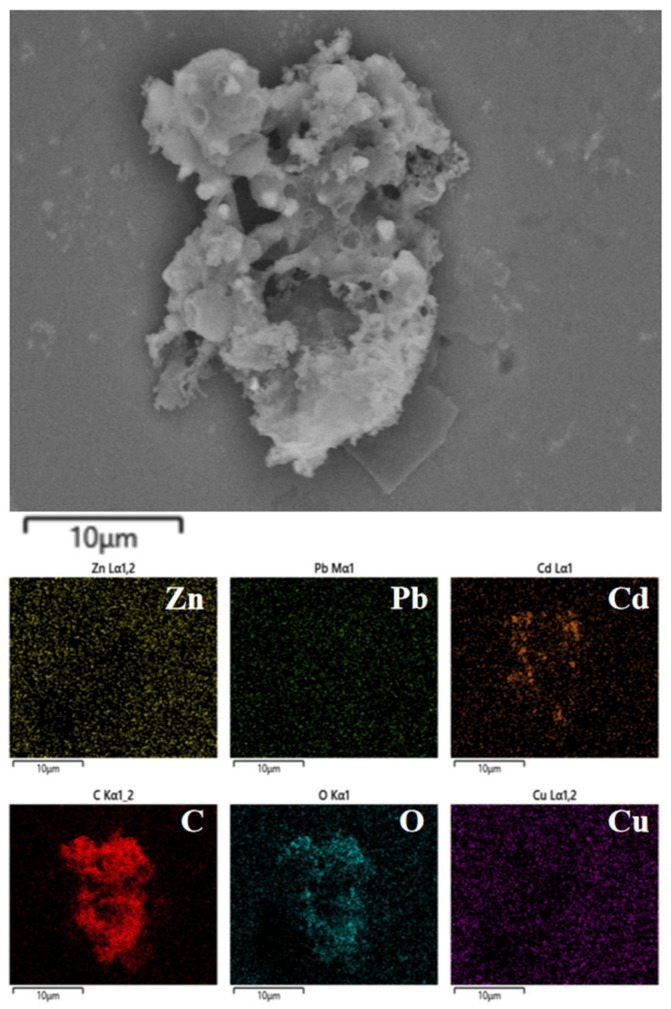
SEM images of different heavy metals.

As shown in Figure 5a, Zn, Pb, and Cd are predominantly enriched in hydrochar (>98%), similar to the migration pattern of Cu. As shown in Figure 6, Zn, Pb, and Cd are homogeneously distributed on the surface of hydrothermal carbon. However, disparities exist in the morphology of the metals. As shown in Figure 5b, no distinct diffraction peaks for Zn are observed. This could potentially be attributed to the formation of complexes between Zn and the surface functional groups or organic matter of the hydrochar during the hydrothermal carbonization process [32]. This complexation may lead to the absence of distinct diffraction peaks due to the lower crystallinity of the complexed zinc or the different crystal structure compared to pure ZnO, ZnS, or other zinc compounds. This complexation reduces the ecological availability of Zn (Figure 5c). As shown in Figure 5c, the ecologically available fractions (F1, F2) of heavy metals Zn, Pb, and Cd are significantly reduced after hydrothermal carbonization. Among these, the ecologically available fractions (F1, F2) of Zn showed the biggest decrease, dropping from 18.7 to 2.5%.

Lead is primarily transformed into the elemental form (2θ ≈ 30.3° (111), 35.1° (200), and 51.6° (220) planes) and carbonate form (2θ ≈ 18.0°, 24.0°, and 32.4°, corresponding to (002), (101), and (102) planes, respectively) during hydrothermal carbonization [33]. The elemental form of lead, due to its low solubility, is fixed in the solid form on the carbon material.

Cadmium primarily exists in elemental form (2θ ≈ 46.5° ((101) crystal plane), 51.2° (corresponding to (102) crystal plane), and 51.6° (corresponding to (110) crystal plane)) and oxidized state (2θ ≈ 26.0°, 32.2°, 46.1°, corresponding to (111), (200), and (220) crystal planes, respectively) during hydrothermal carbonization [34]. Owing to its relatively high solubility and bioavailability, the elemental form of cadmium can form stable complexes through chemical reactions with functional groups on the surface of carbon-based materials during hydrothermal carbonization, thereby reducing its ecological toxicity. The oxidized state of cadmium (such as CdO) is generally more stable, reducing the toxicity of cadmium.

In summary, during the hydrothermal carbonization process, Cu, Pb, and Cd primarily exist in their elemental states, which are generally more stable, thereby reducing the bioavailability and toxicity of these metals. Zn, conversely, mainly exists in a complexed form, potentially forming complexes with surface functional groups of the hydrochar or other metals, thus reducing its toxicity. Pb may additionally exist in a carbonate form, which further decreases its bioavailability and toxicity. Overall, hydrothermal carbonization reduces the ecological toxicity of heavy metals through decreasing their bioavailability. The transformation pathways of various metals during hydrothermal carbonization are closely associated with their physicochemical properties. Due to their metallic characteristics, Cu, Pb, and Cd tend to transform into elemental or carbonate states, exhibiting lower toxicity. Zn, due to its strong hydrophilicity and propensity for complexation, tends to exist in a complexed form, thereby reducing its solubility and bioavailability. In addition, the production of hydrocarbons from biomass contaminated with these heavy metals is economical and cost-effective (Table S2, Supplementary Materials).

## 3. Materials and Methods

### 3.1. Materials and Reagents

The raw biomass used in this experiment was waste pine sawdust (PSD) collected from a furniture factory in Kaifeng, Henan Province, which was used as the model biomass. The main components of pine sawdust include cellulose, hemicellulose, and lignin, with a relatively high carbon content (46.56%) (Table S3), making it suitable for conversion into hydrochar products through hydrothermal carbonization. The collected pine wood was washed with deionized water, and then dried in a drying oven (101-1AB, Beijing Zhongxing, Beijing, China) at 85 °C for 6 h [35]. After drying, the pine wood was crushed using a grinder and sieved through an 80-mesh screen [36]. The sieved material was then placed in a petri dish and further dried in an oven at 85 °C for 12 h before being stored in a sealed bag for use. The reagents, including HNO_3_, HClO_4_, CuSO_4_·5H_2_O, Zn(NO_3_)_2_, Cd(NO_3_)_2_, Pb(NO_3_)_2_, and CH_3_CH_2_OH, were purchased from Anpu Experimental Technology, Shanghai, and Kelong Chemicals, Chengdu, China. All chemicals and reagents were of analytical grade or higher purity and did not require further purification.

In order to accurately study the transformation and effect of heavy metals during the HTC process, heavy metal-contaminated biomass was prepared according to the reported method [37,38]. A certain amount of pine sawdust was added to single metal solutions (CuSO_4_·5H_2_O, Zn(NO_3_)_2_, Cd(NO_3_)_2_, or Pb(NO_3_)_2_) with exact metal ion concentrations, and the mixture was stirred at 200 rpm for 6 h [39]. After adsorption, the mixture was dried at 100 °C to obtain the heavy metal-contaminated biomass (25 or 50 mg heavy-metal/g biomass), which was used for subsequent hydrothermal carbonization experiments.

### 3.2. Hydrothermal Carbonization of Heavy Metal-Contaminated Biomass

**Figure 7:**
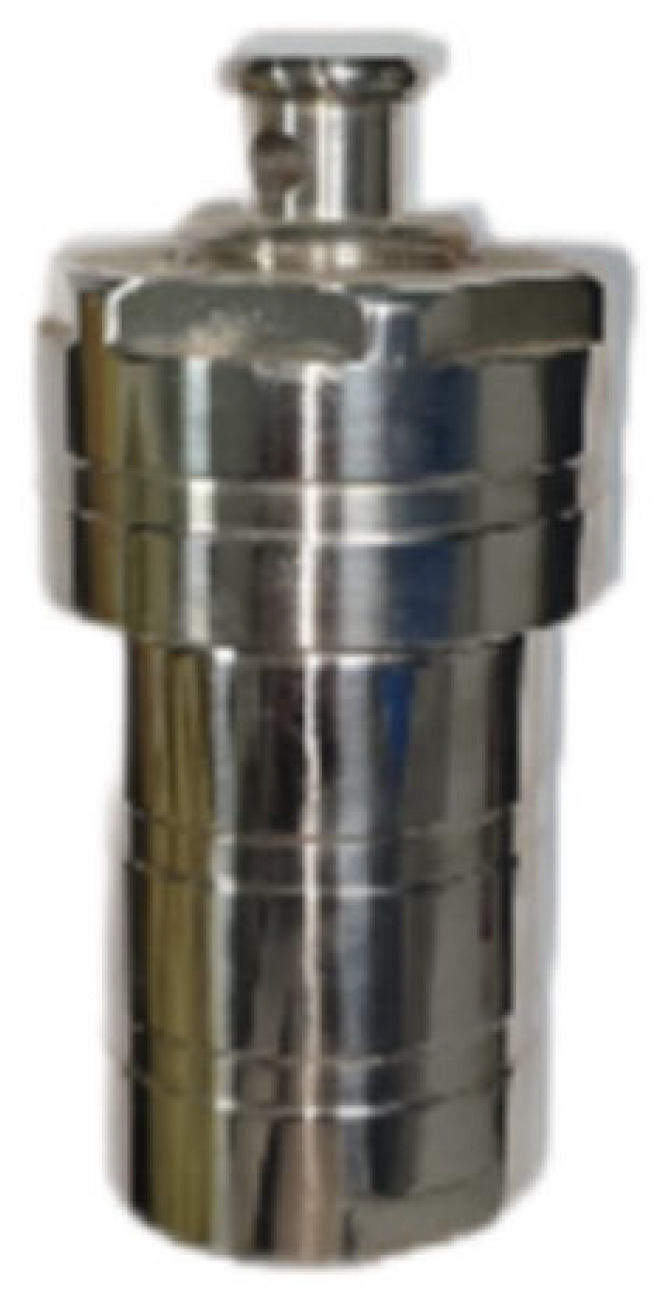
Hydrothermal synthesis reactor (YS-250L, Yushen Instrument, Shanghai, China) used in this study.

An accurately weighed 7.5 g of Cu-PSD (Cu-contaminated PSD) and 75 mL of deionized water were placed into a 250 mL hydrothermal synthesis reactor (Figure 7), and the target temperature and time were adjusted for the reaction. After the reaction was completed, the reactor was naturally cooled to room temperature. The remaining solution in the reactor was filtered to separate the solid and liquid phases using a vacuum pump. The solid phase was washed 2–3 times with deionized water until neutral, then placed into a petri dish and dried in an electric heating air-drying oven at 105 °C for 6 h to complete the preparation. The prepared hydrochar was labeled as “X-T-t-m”, where “X”, “T”, “t”, and “m” represent the heavy metal species, hydrothermal temperature (°C), hydrothermal time (min), and initial heavy metal content (mg/g), respectively.

### 3.3. Characterizations

The microstructure of the hydrochar was observed using a scanning electron microscope (SEM) (Zeiss Merlin Compact, Oberkochen, Germany), which provides high-resolution images of the sample by scanning it with an electron beam. Additionally, mapping scans were performed to examine the elemental distribution in the hydrochar. The elemental compositions of carbon (C), hydrogen (H), nitrogen (N), and oxygen (O) in the samples were analyzed with an elemental analyzer (Vario EL cube, Nidderau, Germany). The phase structure of the samples was determined using an X-ray diffraction (XRD) (Bruker D8 Advance, Karlsruhe, Germany) instrument under conditions of 2θ ranging from 5° to 80°, with a scan rate of 8°/min, 40 mA, and 40 kV. The speciation of heavy metals was analyzed through the Tessier extraction experiment. This procedure classifies heavy metals into five distinct binding fractions: exchangeable fraction (F1), carbonate-bound fraction (F2), iron-manganese oxide-bound fraction (F3), organic matter and sulfide-bound fraction (F4), and residual fraction (F5) [17]. The detailed steps for the Tessier extraction experiment are described in Supplementary Materials. A microwave digestion reactor (BA-SM24, Ba Yue Instrument, Changsha, China) was used to digest the hydrochar by adding a mixture of nitric acid and perchloric acid (volume ratio = 8:2). The volume ratio of nitric acid to perchloric acid typically ranges from 5:2 to 9:1, depending on the sample type and target metal species. For instance, soil and plant samples are commonly digested using a 7:3 ratio (HNO_3_:HClO_4_), while lignocellulosic biomass (e.g., wood or agricultural residues) may require a higher proportion of nitric acid (e.g., 8:2) to fully decompose recalcitrant organic matrices. This optimized 8:2 ratio can provide sufficient oxidation capacity for organic matter breakdown while minimizing potential impurities introduced by excessive perchloric acid. Atomic absorption spectrometer (Beijing Haiguang Instrument Co., Ltd., Beijing, China) was used as the primary instrument to test heavy metal concentration in digested liquids. The concentration of heavy metals in the liquid phase was measured using an atomic absorption spectrometer.

## 4. Conclusions

The migration, transformation, and ecological stability of heavy metals are strongly influenced by the reaction conditions during the hydrothermal carbonization of heavy metal-contaminated biomass. Temperature and heavy metal concentration play crucial roles in altering hydrochar yield and elemental composition. Higher temperatures lead to a decrease in volatile gas production and an increase in stable carbon structures. Copper, zinc, cadmium, and lead exhibit different transformation behaviors, with copper promoting the decomposition of organic materials and enhancing the stability of the carbon product. This study provides valuable insights into optimizing hydrothermal carbonization for effective biomass conversion and heavy metal immobilization. It also provides important foundational data for HTC-based biomass treatment of heavy metal pollution. However, several issues remain to be addressed, such as the long-term stability of heavy metals, the detailed mechanisms of hydrothermal carbonization in fixing heavy metals, and the industrial applicability of the technology. Future research could further optimize this technology through routes like modifying HTC conditions, synergistic pollutant removal, and industrial-scale tests to achieve broader practical applications.

## Figures and Tables

**Table 1:** Changes in yield and elemental composition of hydrochar under different conditions.

Samples	N(%)	C(%)	H(%)	S(%)	O(%) ^a^	O/C ^b^	H/C ^b^	Yield(%)
Cu-170-2-25	1.94	53.31	5.98	0.12	38.65	0.54	1.35	47.54 ^c^
Cu-200-2-25	2.23	57.42	5.16	0.22	34.97	0.46	1.08	26.30
Cu-230-2-0	1.79	57.22	5.73	0	35.26	0.46	1.20	32.29
Cu-230-1-25	2.32	58.24	5.16	0.18	34.10	0.44	1.06	37.94
Cu-230-2-25	1.94	62.27	4.86	0.1	30.83	0.37	0.94	39.35
Cu-230-2-50	1.98	65.06	4.89	0.05	28.02	0.32	0.90	43.35
Cu-230-3-25	2.09	65.79	4.96	0.02	27.15	0.31	0.90	38.13
Cu-260-2-25	1.94	66.43	4.94	0.04	26.66	0.30	0.89	42.53

^a^ Calculated by difference; ^b^ atomic ratio; ^c^ partial carbonization products.

## Data Availability

The original contributions presented in this study are included in the article/Supplementary Materials. Further inquiries can be directed to the corresponding author(s).

## Supplementary Materials

The supporting information can be downloaded at: https://www.mdpi.com/article/10.3390/ijms26062551/s1. References [14,15,17,40,41,42] are cited in the Supplementary Materials.
