# Supplementary material for: Hydrothermal Carbonization of Heavy Metal-Contaminated Biomass: Migration, Transformation, and Ecological Stability Changes of Metals
Source: Int J Mol Sci. 2025 Mar 12;26(6):2551. doi: 10.3390/ijms26062551 (PMC11942420; doi:10.3390/ijms26062551)
Supplement: Supplementary file 1 [file ijms-26-02551-s001.zip › ijms-3464291-supplementary.pdf]

Supporting Information

Number of Pages: 7
Number of Tables: 3

Table S1. Comparison of research on hydrothermal carbonization of various biomass.

Raw material	Research contents	Limitation	Ref.
Water hyacinth	The hydrothermal liquefaction kinetics and product characteristics of copper-impregnated water hyacinth were investigated, with a particular focus on the effects of reaction time on the yields of bio-oil, biochar, aqueous phase products, and gas.	The feasibility and economy of the product in practical applications have not been explored.	[1]
Sludge	The effect of multi-stage process combining hydrothermal carbonization and thermochemical treatment on the migration of phosphorus and heavy metals in sludge was studied Focusing on the migration behavior of phosphorus and heavy metals in sludge treatment.	The experimental conditions are simple and no economic analysis has been conducted.	[2]
Chicken manure and pig manure	Studied on the effects of hydrothermal carbonization at different temperatures on chicken manure and pig manure, with a focus on analyzing the physical and chemical properties, stability, and migration and transformation mechanisms of inorganic components of water char.	No research has been conducted on the migration and transformation mechanisms of heavy metals.	[3]
Sludge and sawdust	Studied on the effect of hydrothermal carbonization conditions on the stabilization of heavy metals in sludge and co-carbonization products, as well as their impact on fuel characteristics.	Lack of in-depth research on the risk of heavy metal release under long-term storage conditions.	[4]
Coking sludge	Explored the migration behavior of heavy metals during the HTC process of coking sludge and evaluated the adsorption capacity of magnetic hydrothermal carbon for heavy metals.	Only specific industrial sludge has been studied, with limited applicability to other types of biomass contaminated with heavy metals.	[5]

Tessier extraction method

Tessier Sequential Extraction Protocol for Metal Speciation Analysis

The Tessier extraction method employs a five-stage sequential chemical extraction procedure to differentiate metal speciation based on their binding characteristics in solid matrices. The operational protocol is detailed as follows:

1. Exchangeable Fraction Extraction

Accurately weigh 1.00 ± 0.01 g of homogenized sample into a 50 mL polypropylene centrifuge tube. Add 8 mL of 1 M magnesium chloride hexahydrate ($MgCl_2 \cdot 6H_2O$) solution. Mechanically agitate the suspension at 200 rpm for 1 h (25 ± 2°C) using an orbital shaker. Separate phases by centrifugation at 4000 × g for 10 min. Decant the supernatant through a 0.45 μm cellulose acetate membrane filter, then quantitatively transfer to a 50 mL volumetric flask and dilute to mark with deionized water.

2. Carbonate-Bound Fraction Extraction

Treat the residual solid from Step 1 with 8 mL of 1 M sodium acetate ($NaCH_3COO$) solution pre-adjusted to pH 5.0 ± 0.1 using glacial acetic acid. Conduct continuous extraction for 8 h at 200 rpm (25 ± 2°C). Centrifuge at 4000 × g for 10 min, filter the supernatant as previously described, and dilute to 50 mL.

3. Iron-Manganese Oxide Fraction Extraction

Add 20 mL of 0.04 M hydroxylamine hydrochloride ($NH_2OH \cdot HCl$) in 25% (v/v) acetic acid solution to the residue. Maintain extraction at 96 ± 3°C using a thermostated water bath with periodic agitation for 4 h. Following centrifugation (4000 × g, 10 min), collect and filter the supernatant, then dilute to 50 mL.

4. Organic Matter-Bound Fraction Extraction

Sequentially add 3 mL of 0.02 M HNO_3 and 5 mL of 30% (v/v) H_2O_2 to the residue, adjusting to pH 2.0 ± 0.1 with concentrated HNO_3. Heat the mixture to 85 ± 2°C for 2 h with intermittent shaking. Add additional 5 mL H_2O_2, readjust pH to 2.0, and continue heating at 85 ± 2°C for 3 h. After cooling to ambient temperature, introduce 5 mL of 3.2 M ammonium acetate (NH_4CH_3COO) and dilute to 20 mL with 20% (v/v) HNO_3. Agitate for 30 min, centrifuge, filter, and adjust to final volume.

5. Residual Fraction Extraction

Quantitatively transfer the final residue to a PTFE digestion vessel using 10% HNO_3. Evaporate to near dryness (≈3 mL residual volume) on a hotplate. Add 15 mL concentrated HNO_3, 10 mL HF, and 5 mL $HClO_4$. Digest sequentially at 180°C until cessation of white fumes ($HClO_4$). For incomplete digestion, repeat with 5 mL HF additions until obtaining a

white/light yellow residue. Dissolve the residue in 0.5% HNO_3 with gentle heating, cool to room temperature, and transfer quantitatively to a volumetric flask for analysis.

Table S2. Cost estimation of 1 kg hydrochar production from heavy metal-contaminated biomass (dry weight).

Particulars	Sub-sections	Cost break up	Total cost (USD)
**First Step**			
**Raw material processing**	Washing Cost	Electricity cost for preparing 1 L of deionized water<br>hour × unit × per unit cost = 1 × 0.5 × 0.058	0.029
	Drying Cost (85 °C)	hours × unit × per unit cost = 6 × 1 × 0.058	0.348
	Size reduction cost	The size reduction was done through a crusher<br>hour × unit × per unit cost = 0.2 × 1 ×0.058	0.012
**Second Step**			
**Preparation of biochar**	Carbonization Cost (Carbonization at 230 °C)	hours × unit × per unit cost = 2 × 1 × 0.058	0.116
	Washing Cost	Electricity cost for preparing 1 L of deionized water<br>hour × unit × per unit cost = 1 × 0.5 × 0.058	0.029
	Drying Cost (105 °C)	hours × unit × per unit cost = 6× 1 × 0.058	0.348
**Net Cost**			**0.882**
**10% of overall cost**			**0.088**
**Total cost**			**0.97**

Here, the cost calculation is performed by considering the electricity charge per unit consumption to be 0.058 USD.

The estimated cost is comparable to the price of the commercial carbon material from biomass (1-5 USD) [6].

Table S3. Proximate and elemental characterization of the PSD biomass.

Proximate analysis (wt.%)		Elemental analysis (wt.%)	
moisture	4.21	C	48.56
volatile	78.34	H	5.56
fix carbon	15.88	N	0.21
ash	1.57	O [a]	45.67
		S [b]	< 0.005

[a] calculated by difference (O = 100-C-H-N);

[b] determined by ICP-AES

Reference

1. Yadav, P.; Reddy, S.N. Reaction kinetics for hydrothermal liquefaction of Cu-impregnated water hyacinth to bio-oil with product characterization. *Industrial Crops and Products* **2023**, *198*, doi:10.1016/j.indcrop.2023.116677.
2. Stobernack, N.; Malek, C. hydrothermal carbonization combined with thermochemical treatment of sewage sludge: Effects of $MgCl_2$ on the migration of phosphorus and heavy metal. *Waste Management* **2023**, *165*, 150-158, doi:10.1016/j.wasman.2023.04.010.
3. Zhang, J.; Wang, Y.; Wang, X.; Wu, W.; Cui, X.; Cheng, Z.; Yan, B.; Yang, X.; He, Z.; Chen, G. Hydrothermal conversion of cd/zn hyperaccumulator (*sedum alfredii*) for heavy metal separation and hydrochar production. *Journal of Hazardous Materials* **2022**, *423*, doi:10.1016/j.jhazmat.2021.127122.
4. Lu, X.; Ma, X.; Qin, Z. Co-Hydrothermal Carbonization of Sewage Sludge with Wood Chip: Fuel Properties and Heavy Metal Transformation Behavior of Hydrochars. *Energy & Fuels* **2021**, *35(19)*, 15790-15801, doi:10.1021/acs.energyfuels.1c02145.
5. Zhong, J.; Yan, X.; Wu, C.; Wu, Y.; Zhang, H.; Bu, Y. Hydrothermal carbonization of coking sludge: Migration behavior of heavy metals and magnetic separation performance of hydrochar. *Journal of Environmental Chemical Engineering* **2024**. *12(5)*, doi:10.1016/j.jece.2024.114141.
6. Zhang, F.; Wang, J.; Tian, Y.; Liu,C.; Zhang S.; Cao, L. Effective removal of tetracycline antibiotics from water by magnetic functionalized biochar derived from rice waste. *Environmental Pollution* **2023**, *330*, doi:10.1016/j.envpol.2023.121681.
